# Education as an upstream social security intervention: constructing a competency-based talent development framework in health economics

**DOI:** 10.3389/fpubh.2026.1782959

**Published:** 2026-02-26

**Authors:** Haohan Luo, Ailin Li, Haiyan Hua

**Affiliations:** Business School, Chengdu University of Technology, Chengdu, China

**Keywords:** competency-based education, health economics, health policies, social security, talent development, upstream intervention

## Abstract

Confronted with structural challenges arising from an aging population, rising chronic disease burdens, and inequitable resource allocation, traditional downstream interventions are increasingly inadequate for effective health governance. This study argues that the critical bottleneck lies in a “human capital deficit,” where current education fails to equip students with interdisciplinary knowledge and competency to solve complex real-world challenges. To address this, we propose an upstream social security intervention by establishing a competency-based talent development framework in health economics. We construct a “T-shaped” talent development model that integrates systemic vision with professional depth. The framework operates on three levels: (1) Principles: Adopting principles of demand orientation, competency drive, and systems thinking to establish a comprehensive curriculum paradigm integrating knowledge, skills, and professional attributes; (2) Pedagogy: Implementing outcome-based education (OBE), real-world simulation-based teaching, and progressive case study method to bridge the gap between theoretical modeling and real-world policy implementation; and (3) Mechanism: Establishing a collaborative development mechanism among the government, industry, universities, and research institutions to ensure the dynamic alignment of talent competencies with evolving social security demands. Positioning the framework as a strategic upstream intervention, this study aims to cultivate health economists capable of applying practical competency and interdisciplinary knowledge to address complex health challenges, ultimately offering a sustainable pathway to enhance the governance effectiveness and equity of social security systems.

## 1 Introduction

In recent years, driven by profound transformations in the global socio-economic landscape, healthcare reform and health policy adjustments have extended beyond the traditional boundaries of the health sector, emerging as a critical issue concerning the sustainability of social security systems. Irrespective of developmental stage or institutional design, social security systems worldwide are confronting a series of converging structural challenges that severely test their financial solvency, equity, and governance effectiveness.

These structural challenges manifest primarily in three dimensions. First, population aging is pervasive globally. The United Nations (UN) projects that by 2050, the global population aged 65 and above will reach 1.6 billion, accounting for 16.33% of the total population.^1^ This demographic shift is driving a continuous rise in the prevalence of non-communicable diseases (NCDs), which are gradually replacing traditional infectious disease patterns (2). Older adults generally suffers from multimorbidity, necessitating complex and prolonged healthcare, thereby imposing unprecedented pressure on healthcare delivery, pension expenditures, medical insurance solvency, and long-term care systems (1). Second, the burden of chronic diseases accompanying population aging continues to intensify. The World Health Organization (WHO) reported that in 2021, NCDs—represented by cardiovascular diseases, cancer, chronic respiratory diseases, and diabetes—caused at least 43 million deaths.^2^ The WHO predicts that the rapid escalation of NCDs will not only surge household healthcare expenditures but also impede poverty alleviation efforts in low-income countries. Third, the inequitable allocation of health resources and associated efficiency losses undermine the principle of universality in social security. In both developed and developing nations, the phenomenon known as the Inverse Care Law—wherein those most in need of medical services are least likely to receive them—remains a prevalent issue (3, 4). High-quality medical personnel, advanced medical technologies, and financial resources are commonly concentrated in large hospitals within central cities. This results in a low effective benefit ratio for grassroots beneficiaries, weakening overall efficiency of the social security system and exacerbating disparities in social security benefits among different population groups.

These global structural challenges are particularly pronounced and complex within the context of China. By the end of 2024, China’s population aged 65 and above surpassed 220 million, accounting for 16% of the total population, with the old-age dependency ratio reaching 22.8%.^3^ This signifies that China, the country with the largest population, has entered a critical phase in addressing the challenge of advanced aging. Such profound population aging necessitates increasingly refined management of China’s massive basic medical insurance and pension insurance funds. WHO statistics reveal that as of 2021, NCDs accounted for 90.7% of all deaths in China.^4^ The structural challenges encountered by China further underscore the universal dilemmas faced globally in constructing efficient social security systems.

Confronted with these structural challenges arising from an aging population, rising chronic disease burdens, and inequitable resource allocation, existing policy instruments and reform pathways have demonstrated notable limitations. Investigating the underlying common cause reveals a critical bottleneck: human capital deficit. Specifically, there is a serious scarcity of interdisciplinary professionals capable of systematically integrating knowledge from economics, management, medicine, law, and other related fields. The educational paradigm dominated by a single discipline in the current educational system has caused graduates to be inadequate in addressing public health challenges, often leading to shortsighted and inefficient policy formulation.

Human capital is a critical factor in maintaining the long-term solvency and service efficiency of social security systems. To address this, we introduce the theory of upstream intervention. This theory is renowned for a classic metaphor: If people keep drowning downstream, and we only carry out emergency rescue downstream without investigating why they fall in upstream, the problem will persist indefinitely (5). Downstream interventions focus on addressing public health issues that have already manifested and are inherently treatment-centric. Hospitals primarily function as these downstream rescuers. However, evidence suggests that downstream interventions may inadvertently exacerbate health inequities among socio-economic groups, as they often confer greater benefits on already advantaged populations (6). The upstream intervention perspective necessitates a shift in focus toward root causes. When addressing challenges such as an aging population, rising chronic disease burdens, and inequitable resource allocation, a fundamental question arises: beyond specific measures, do we possess the health economics talent capable of navigating the complexities of future social security systems? Developing health economics talent equipped with interdisciplinary knowledge in economics, management, medicine, law, and regulations directly responds to the urgent social demand for interdisciplinary public health talent.

Health economics concerns issues of efficiency, effectiveness, value, and behavioral patterns during the production and consumption of health services and healthcare. It investigates how to rationally allocate finite resources to enhance the inclusivity and governance effectiveness of social security (7). By utilizing economic theories, economic models, and empirical techniques to analyze decisions about health and healthcare, health economics is dedicated to resolving the most pressing challenges facing health systems (8). From the perspective of endogenous growth theory, education serves as a fundamental, long-term investment in human capital. The new generation of health economists fostered by this intervention is poised to become the critical human capital for improving health governance. Consequently, establishing a competency-based talent development framework in health economics represents a prospective, fundamental, and strategic intervention in both social security and education.

This study provides higher education with a competency-based talent development framework that combines theoretical depth with practical feasibility in relevant discipline development and curriculum construction. It aims to cultivate health economics talent possessing both practical competency and interdisciplinary knowledge to address complex health challenges and enhance the governance effectiveness and equity of social security systems. The remainder of this paper is organized as follows: First, we analyze the necessity of establishing this framework and propose the guiding principles for discipline development and a “T-shaped” talent development model. Then we elaborate on the core content of this framework, including a comprehensive curriculum paradigm and innovative pedagogical methods. Subsequently, we explore an effective path to implement the framework and establish a collaborative development mechanism among the government, industry, universities, and research institutions. Finally, a comprehensive discussion and concluding remarks are presented.

## 2 Necessity and strategic design: developing a new generation of health decision-makers

Addressing the structural challenges within the social security system fundamentally requires an upstream intervention through innovative educational reform. Specifically, this entails the systematic development of interdisciplinary health economics professionals who can empower future social security systems. Based on literature review and expert consultation, we propose a “T-shaped” talent development model. Furthermore, we delineate guiding principles for discipline development to establish a solid theoretical foundation for a concrete curriculum paradigm.

### 2.1 Urgency analysis: limitations of current health economics talent development

The intrinsic complexity of health issues determines the interdisciplinary nature of health economics. Health economics is not merely the economics of studying input factors in medical policy, but also the economics of analyzing health behaviors and services (9, 10). A review of global higher education, particularly in transition economies, reveals a significant structural mismatch between talent supply and actual demand. Investigating the root cause, the entrenched cognitive barriers resulting from a single-disciplinary paradigm hinder the development of students’ systems thinking. This limitation impairs their ability to effectively address complex challenges in the real world.

Within the current education framework, the interdisciplinary essence of health economics is inadequately integrated into university curricula. The fragmentation of teaching content remains pervasive, and a general lack of holistic curriculum design prevents students from developing a comprehensive and systemic disciplinary perspective (11, 12). In medical schools, education remains confined to clinical diagnosis and treatment techniques. Medical students often lack awareness of the accessibility, scarcity, and incentive mechanisms of health resources, failing to grasp the economic logic underpinning macro policies. In schools of economics and management, teaching emphasizes macroeconomic and microeconomic theories and the deduction of economic models. Students often lack an intuitive understanding of disease history, public health interventions, and medical ethics, resulting in models they built being divorced from clinical practice. This disciplinary silo leads to a deficiency in the composite knowledge and competence required to address complex health challenges.

This fragmented educational landscape makes it difficult for students to achieve maximum health outcomes with limited public health resources. The dilemma between balancing epidemic control efficacy against economic cost and technological advancement against insurance affordability in various countries during the COVID-19 pandemic (13), along with the frequent policy patches in medical insurance payment reform (14, 15), demonstrates the imperative need to establish a competency-based talent development framework in health economics.

### 2.2 Interdisciplinary talent development model: “T-shaped” knowledge structure and core competencies

Single-discipline-oriented “I-shaped” talent is no longer sufficient to cope with increasingly complex public health challenges. The competency-based talent development framework in health economics proposes a “T-shaped” talent development model, fostering professionals with both broad cognitive horizons and specialized depth.

The horizontal dimension of the “T-shaped” talent development model represents broad interdisciplinary knowledge and systemic vision, serving as the prerequisite for implementing upstream interventions. This dimension requires students to transcend disciplinary boundaries among economics, management, medicine, law, and other related fields. Beyond mastering the knowledge of foundational medicine and public health, students should comprehend how relevant factors, such as education, income distribution, social security systems, and public health policies, influence population health and the efficiency of social security through complex transmission mechanisms. This horizontal integration of knowledge equips professionals with a macro-level and systemic perspective to consider health issues.

The vertical dimension of the “T-shaped” talent development model signifies deep specialization within a specific domain upon a base of broad interdisciplinary knowledge. It requires students, after developing a systemic perspective, to anchor themselves in a core field—such as pharmaco-economics or healthcare security systems—for vertical knowledge construction and skills acquisition. Through the precise mastery of advanced tools, students acquire the capacity to translate theoretical models into evidence-based practice, thereby applying rigorous empirical methods to resolve difficulties in resource allocation.

### 2.3 Guiding principles for discipline development: demand-orientation, competency drive, and systems thinking

To ensure the effective operation of the “T-shaped” talent development model, the framework establishes three guiding principles. These principles form the bedrock of the framework, comprehensively guiding the entire process from curriculum design to outcomes assessment.

#### 2.3.1 Demand orientation

Demand orientation necessitates that the talent development in health economics transcends academic silos and actively aligns with national strategic needs and frontiers of the health industry. Whether the commitment to “Good Health and Well-being” in the United Nations Sustainable Development Goals (SDGs) or national health strategies formulated to address population aging, the burden of chronic diseases, and public health emergencies, these constitute the top-level strategic context for talent development. Simultaneously, as the health industry transitions from marketing-driven to value-driven models, market demands from pharmaceutical enterprises, commercial health insurers, and digital healthcare sectors should serve as guiding indicators for talent development (16).

#### 2.3.2 Competency drive

This principle emphasizes a shift from traditional knowledge transmission to Competency-Based Education (CBE). In an era of information explosion, knowledge updates rapidly, whereas core competencies possess enduring transfer value (17). The competency encompasses not only technical skills in applying economic models for analysis, but also the ability to make value judgments in complex contexts, advocate for policy, and engage in evidence-based decision-making grounded in big data. The contemporary pedagogical objective of health economics has evolved from the rote memorization of economic theories or medical terminology to the development of comprehensive capabilities. This empowers students to rapidly synthesize interdisciplinary knowledge, construct analytical frameworks, and formulate actionable solutions for complex public health challenges.

#### 2.3.3 Systems thinking

Systems thinking serves as the underlying logic of the framework. The public health system is an inherently Complex Adaptive System (CAS) characterized by high dynamism, openness, and interactivity (18). As a methodology for addressing problems from a holistic perspective, systems thinking focuses on the interconnections among the components of a system. Moreover, applying systems thinking to health systems has been proven beneficial. As Trochim et al. noted, introducing systems thinking into health systems holds significant practical value for constructing a panoramic view of financing, deepening interdisciplinary collaborative mechanisms, and addressing the influence of social and political factors (19, 20). Systems thinking enriches the theories, methods, and instruments available to health economics professionals in global health and social security governance. It provides new opportunities to comprehend, iteratively test, and revise the understanding regarding the essence of health issues (21), thereby facilitating the provision of more resilient and sustainable solutions for complex health governance.

## 3 Core content of the development framework: integration of curriculum, methodology, and practice

Guided by the principles of demand-orientation, competency drive, and systems thinking, the core content of the framework is structured around a central objective: achieving a profound integration of theoretical knowledge, professional skills, and professional attributes. This integration is designed to be internalized by students, equipping them with the comprehensive capacity to address complex health challenges in the real world. To achieve this, the framework proposes a talent development scheme comprising a comprehensive curriculum paradigm and innovative pedagogical methods.

### 3.1 Curriculum content: a comprehensive curriculum paradigm

While maintaining a strong emphasis on general education, health economics talent development should adhere to the Competency-Based Education by integrating knowledge, competencies, and professional attributes into a coherent whole. This ensures students concurrently accumulate knowledge, enhance practical abilities, and cultivate professional ethics throughout their learning process.

#### 3.1.1 Knowledge dimension

Given the interdisciplinary nature of health economics, the competency-based talent development framework in health economics adopts a “T-shaped” talent development model, dividing the knowledge into two components: the “Foundational General Education Modules” and the “Specialized and Refined Direction.” The Foundational General Education Modules comprise three core modules. Module 1: Economic Principles and Behavioral Health. This module covers microeconomic concepts such as resource scarcity and incentive mechanisms, as well as macroeconomic topics including health capital and economic growth. Specifically, behavioral health economics is incorporated to explore the application of bounded rationality, time preference, and nudge theory in chronic disease prevention and healthy lifestyle interventions (22). This module emphasizes economic analysis and causal inference, fostering evidence-based thinking grounded in data. Module 2: Medical Foundations and Public Health Science. Covering epidemiology, health statistics, social medicine, and health services management, this module prioritizes the integration of the whole-life-cycle health management. And it enables students to comprehend the long-term cost–benefit logic of transitioning from disease treatment to health promotion within an aging population context. Module 3: Global Health Systems and Policy Regulation. Utilizing a comparative health systems perspective, this module examines financing models, health service systems, and governance structures across different nations in their pursuit of universal health coverage. It focuses on the social determinants of health inequalities, developing students’ global perspective.

Building upon a broad interdisciplinary foundation, students should select specific fields for vertical specialization based on their career planning. Four specialized and refined directions are defined according to industry demands and disciplinary frontiers. Direction 1: Health Technology Assessment (HTA) and Pharmacoeconomics. This direction develops students’ capacity to conduct economic modeling and value assessments using clinical trial data and real-world evidence. Students should engage with advanced courses such as cost-effectiveness analysis, cost–benefit analysis, and budget impact analysis to learn how to optimize the market access and pricing of innovative medical technologies under budget constraints. Direction 2: Medical Security and Strategic Purchase. This direction focuses on how payers can induce behavioral changes in healthcare providers through strategic purchasing. Students are required to undertake advanced courses in payment system design, actuarial principles, and risk adjustment mechanisms to enhance the efficiency of health fund utilization. Furthermore, the curriculum incorporates Game Theory to enhance students’ strategic negotiation competencies. Direction 3: Health Industry Innovation and Management. This direction explores how to balance the economic returns and social benefits of the health industry under market mechanisms. Students are required to further undertake advanced courses such as healthcare operations management and value chain analysis of the health industry. These courses are designed to develop their competencies in dealing with the “silver economy” and the upgrading of health consumption. Direction 4: Digital Health and Big Data Governance. This direction explores health governance and evaluation in the context of digital transformation. Students are required to study the application of medical big data analytics and machine learning in health economics, economic evaluation of digital therapeutics, data privacy, and ethical regulation (23, 24).

To ensure the effective implementation of the above “modular and refined” knowledge structure, universities can implement a micro-credential study scheme. Characterized by the compact and structured format, micro-credential facilitates the effective integration of interdisciplinary knowledge at relatively low opportunity cost (25). For instance, medical students may make up for shortcomings in the resource allocation perspective by taking an economics micro-credential, while business and management students may deepen their understanding of chronic disease burdens and the long-term sustainability of health resources through a public health micro-credential. The micro-credential study scheme must incorporate rigorous assessment standards and credit certification mechanisms equivalent to core curriculum requirements, ensuring it serves as a substantive component of the talent development framework rather than a mere supplement.

#### 3.1.2 Skills dimension

The skills dimension centers on efficiently translating interdisciplinary knowledge into practical capabilities for resolving complex health challenges. Relevant skills can be summarized into four aspects. First, the capability for data acquisition and analysis. Contemporary health economic decision-making increasingly relies on big data and evidence-based analysis (26). Students should be proficient in acquiring, cleaning, and processing health data, and effectively integrate medical insurance databases, hospital information systems, and epidemiological survey data. Given the sensitivity of health data, students must strictly adhere to ethical standards and data compliance regulations during data mining and processing. On this basis, priority is given to developing core competencies in applying economic analysis tools for policy evaluation and empirical reasoning. Second, the capability for economic modeling and resource allocation. Health economics professionals should systematically integrate economic theory, medical information, and policy regulations to construct resource allocation models tailored to practical health needs. They are required to design resource allocation schemes under budgetary constraints by comprehensively balancing multiple objectives, including clinical effectiveness, health outcomes, economic feasibility, and equity in social security (27). Third, the capability for policy design and implementation. A competent health economic professional can not only propose theoretical solutions but also implement them precisely. This feature includes policy design abilities as well as the capacity to promote policy execution in multi-departmental and multi-stakeholder settings, coordinate varied stakeholder interests, and manage dynamic changes and risks. Fourth, the capability for cross-disciplinary communication and collaboration. Health economics issues involve multiple fields, including economics, management, medicine, law, and public health. Addressing them depends on teamwork and cross-sectoral coordination. Students should develop strong communication skills, empathy, and social responsibility, enabling them to establish evidence-based dialog across disciplines and departments to promote policy implementation and health equity.

#### 3.1.3 Professional attributes dimension

The professional attributes dimension constitutes the intrinsic foundation for talent development. Its essence lies in internalizing values, professional ethics, and social responsibility as behavioral guidelines. This directs professionals to consistently uphold a human-centric awareness and enhance societal well-being while applying their knowledge and skills. Only under the guidance of solid values, professional ethics, and social responsibility can the knowledge and skills of health economics be transformed into effective forces that promote health equity and the effectiveness of social security. The professional attributes dimension first demands the firm establishment of health equity awareness. Health economics not only pursues the efficiency of resource allocation but also strives to enhance health equity and sustainability. Consequently, the framework must deeply embed health equity concepts into curricula. Secondly, adhering to the baseline of professional ethics is crucial. Students are expected to consciously respect boundaries among medicine, humanity, and law. When confronted with situations involving patient privacy, ethical dilemmas, and the spillover effects of policies, they should consistently uphold basic principles such as the protection of patients’ rights and informed consent (28). Finally, developing professional attributes relies on a strong sense of social responsibility. Students should be encouraged to integrate their personal development goals with the broader vision of global health. They are expected to actively participate in healthcare services at the grassroots level and in remote areas, contributing to the equitable and inclusive development of healthcare systems.

### 3.2 Innovative pedagogical methods: bridging theory and practice

To bridge the gap between theoretical cognition and real-world cases, the framework must adopt methods that transcend traditional knowledge transmission. The framework requires establishing an innovative pedagogical paradigm anchored in Outcome-Based Education (OBE) and supported by real-world simulation-based teaching and a progressive case study method. The paradigm aims to transform disciplinary knowledge into practical capabilities that optimize medical resource allocation, enhance the quality and efficiency of healthcare, and promote the long-term sustainability of social security.

#### 3.2.1 Outcome-based education

In the development of health economics professionals, the traditional “input-driven” teaching method is increasingly inadequate for addressing complex and evolving public health challenges. OBE focuses on competency outputs, directly aligning students’ academic achievements with the demands of national strategies, social security, and the health industry. By constructing “competency profiles” for students, higher education institutions can restructure curricula and instructional processes. OBE emphasizes backward design, taking students’ future competencies as the guiding direction. First, the core competencies required for key positions in the field of health economics should be systematically mapped to determine the capabilities graduates must possess. Subsequently, in accordance with OBE requirements, break these competencies into quantifiable learning objectives and further cascade them into distinct course modules. Simultaneously, attention must be paid to evolving societal health demands, industrial transformations, and new policy regulations. In response to these dynamics, universities should timely adjust course content, refresh the competency list, and optimize pedagogical and assessment methods. Health economics is an applied and interdisciplinary field. OBE can effectively integrate knowledge, skills, and professional attributes. It organizes the entire talent development chain around the strategic demands of the social security system, injecting sustainable momentum into future health economists to solve real-world health problems (29, 30).

#### 3.2.2 Real-world simulation-based teaching

Public health policies typically involve vast population scales and significant capital volumes, characterized by pronounced externalities and irreversibility (31, 32). Traditional class instruction often fails to enable students to grasp the potentially catastrophic consequences of a flawed policy design. Therefore, the framework introduces real-world simulation-based teaching, allowing students to engage in policy experimentation and optimization in a risk-free environment. This method centers on topical issues such as health insurance payment reform, hierarchical diagnosis and treatment, innovative drug access, and structural reforms in the health industry, emphasizing the simulation of the strategic interactions among multiple stakeholders in health decision-making. This pedagogical approach brings authentic situations into the classroom. It employs role-play and structured debate to position students at the contradictory center of health issues. In these scenarios, students are grouped to simulate stakeholders, such as patients, doctors, hospital managers, medical insurance bureau officials, and pharmaceutical company representatives, unfolding work around real-world health topics. For example, whether high-priced innovative drugs should be included in medical insurance. The simulation requires students to utilize data analysis and economic models to generate evidence, and engage in argumentation and negotiation during the final simulated policy hearing. A big data-based simulation laboratory should incorporate multi-source heterogeneous data that is desensitized, such as medical insurance settlements, hospital services, population mobility, chronic disease burden, and public health statistics. Students can independently set key parameters, including reimbursement rates, payment methods, medical treatment procedures, drug prices, and insurance coverage. Subsequently, the system conducts real-time policy simulations and provides dynamic feedback on outcomes, including medical insurance fund balance, patient flow, population health indicators, and service accessibility. Students then iteratively refine policies based on feedback information. This iterative cycle of “parameter input—outcome feedback—policy adjustment” develops students’ competencies in assessing policy time lags, marginal impacts, and systemic risks.

#### 3.2.3 Progressive case study method

As a practice-oriented teaching methodology, the case study method has demonstrated remarkable educational effectiveness in enhancing students’ critical thinking, analytical reasoning, and practical skills (33). However, the traditional case study method lacks coherence and systematicity, remaining confined to a single-discipline perspective and failing to meet the educational demands for interdisciplinary knowledge application. The framework proposes a progressive case study method. This approach fosters students’ composite analytical capabilities by embedding systematic, progressive, and multidisciplinary integrated cases throughout the curriculum cycle. The innovation and effectiveness of the method lie in engaging students in iterative and progressive analysis of a single case, gradually equipping them with the competency to address complex problems in public health systems. Each stage of case analysis provides dynamic feedback on prior outcomes, fostering students’ forward-thinking capabilities regarding policy implementation consequences. This method is helpful to equip students with a robust foundation for professional roles in health resource allocation, social security system design, and evaluation. Throughout this process, students analyze selected cases from multi-disciplinary perspectives, including economics, management, medicine, law, and policy regulation. The complexity of cases increases in alignment with course progression. To ensure effectiveness, cases selected must be challenging and open-ended. They should reflect the interdisciplinary characteristics of health economics and align with different curriculum stages. In addition, priority should be given to recent cases, ensuring students engage with the latest changes in the health industry and public health policy.

## 4 Implementation pathways and guarantee mechanism

To effectively facilitate the high-quality development of interdisciplinary talent in health economics, it is imperative to construct a collaborative development mechanism involving the government, universities, industry, and research institutions. This mechanism is government-guided, university-anchored, and actively supported by the health industry and research institutions. The collaborative development mechanism serves not only as a conduit for knowledge transmission but also as a carrier for skills enhancement and a platform for value co-creation. It can effectively align health economics talent development with national social security strategies and the development of the health industry.

### 4.1 The operational mode of the collaborative development mechanism

Within this collaborative ecosystem, government, universities, industry, and research institutions collectively promote innovation and development in health economics through strategic communication and cooperation. The government functions as a top-level architect, providing policy guidance and funding support to ensure the alignment of talent development with macroeconomic health policies. Universities undertake the core education responsibility, focusing on the construction of knowledge, the promotion of scientific research, and the development of core competencies. The health industry provides scenarios for practice and technical application, co-establishing “university-enterprise” practice bases with universities and participating in the entire curriculum design and development process. Furthermore, industry partners provide feedback on employment standards and assess students’ practical skills and market adaptability. Research institutions undertake major research projects, provide high-quality data resources, and develop cutting-edge theories and technical tools. To ensure the efficient operation of this collaborative development mechanism, a “Joint Committee for Health Economics Education” should be established, comprising representatives from all stakeholders. The committee is mandated to formulate talent development standards, participate in teaching resource allocation, and oversee the quality of the collaborative development mechanism across all stages to ensure the sustainability of the mechanism. The committee’s core functions include: (1) Standard formulation: Jointly defining competency frameworks, internship assessment criteria, and project evaluation standards. (2) Resource coordination: Facilitating resource allocation and information sharing among collaborative development participants. (3) Supervision and management: Strengthening management, service, and supervision throughout the entire collaborative process to ensure orderly operations. (4) Quality assessment: Regularly evaluating implementation status, fund utilization, and educational outcomes to continuously optimize cooperation models.

### 4.2 Key collaborative models

#### 4.2.1 Collaborative curriculum development and dual-teacher instruction

This model disrupts the traditional paradigm where university faculty are the sole source of professional knowledge by inviting experts from the government, industry, and research institutions to participate in course design, case development, teaching, and evaluation. Prior to course development, academic faculty and external experts should jointly identify target competencies the course aims to cultivate. Based on national strategies and critical industry challenges, real-world policy cases and cutting-edge technologies should be integrated into instruction. During instruction, both internal and external faculty should jointly undertake lecturing, case seminars, student guidance, and performance assessment. University faculty systematically present health economics theoretical frameworks and analytical tools, while industry and government experts enrich the learning experience with practical case analyses, practical operations, policy interpretations, and project-based expertise (34, 35). This model achieves a tripartite integration of “disciplinary knowledge—industry scenarios—policy hotspots.” Students can transform abstract academic models into concrete tools and solutions for navigating market and policy transformations.

#### 4.2.2 Co-construction of practice bases and the dual-mentor system

This initiative aims to transform practical training from short-term internships into institutionalized, long-term practice integrated throughout the entire educational process. This model goes beyond simple nominal collaboration bases, emphasizing joint resource investment by universities and partner organizations, such as local medical insurance bureaus, leading medical groups, and health technology firms. Students participate as formal team members in authentic projects. The establishment of joint bases signifies a shift from a mere talent supply relationship to a strategic partnership, where collaborators jointly invest in, manage, and cultivate health economics professionals (36, 37). Each student is assigned an academic mentor from the university and a practical mentor from the external partner, forming a dual-mentor system with shared responsibility. Academic mentors ensure scholarly rigor and guide students in identifying researchable health issues from specific tasks. Practice mentors assign challenging real-world projects and guide students in mastering industry standards and workflows.

#### 4.2.3 Joint research projects and data collaboration

To deeply integrate innovation capabilities with research output, universities should collaborate with government agencies, the health industry, and research institutions to undertake research projects. These projects should be oriented toward addressing real-world challenges such as population aging, the burden of chronic diseases, and health equity. This approach not only hones students’ research skills but also directly serves practical needs for enhancing population health and social security. As research assistants, students are immersed in project design, execution, and reporting, realizing a mode of “research-driven learning.” To address the critical difficulty of data accessibility in health economics, stakeholders should facilitate the establishment of secure, compliant, and shared databases. For instance, universities and their partners could jointly develop a “Health Economics Data Collaboration Platform” to integrate multi-source, heterogeneous information such as regional medical insurance settlements data, public health monitoring data, chronic disease management data, and healthcare service databases. Data providers should utilize de-identification and encryption technologies to desensitize datasets, strictly preventing the re-identification of individuals.

### 4.3 Quality assurance and continuous improvement mechanisms

To ensure the long-term operation of the collaborative development mechanism, it is essential to establish a quality assurance mechanism that is data-driven, multi-party participatory, and adaptive. This mechanism evaluates student learning outcomes while continuously validating and refining the effectiveness of the entire upstream intervention strategy.

#### 4.3.1 Multi-dimensional teaching quality assessment mechanism

To accurately measure educational outcomes of the competency-based talent development framework in health economics, universities should establish a multi-dimensional teaching quality assessment mechanism. This mechanism aims to make implicit competencies explicit, ensuring that assessment results authentically reflect students’ ability to address real-world health economic problems. The mechanism emphasizes students’ growth trajectories rather than solely focusing on learning outcomes. (1) Introduce a learning portfolio to document the performance of students across classroom learning, innovative pedagogical methods, and collaborative development mechanism. (2) Drawing competency radar charts to visualize students’ performance across core competency dimensions, including critical thinking, interdisciplinary collaboration, and ethical decision-making. By comparing these profiles in different educational phases, the trajectory of students’ competency development is monitored, serving as a pivotal metric for evaluating pedagogical effectiveness. (3) Construct a dual-track assessment model combining academic and practical components. The academic module emphasizes theoretical mastery and methodological rigor, while the practical module evaluates students’ performance in real-world projects. (4) To examine the actual contribution of the framework to social security systems, it is necessary to add long-term impact evaluation indicators, such as policy contributions and cost-saving initiatives implemented by graduates in their future careers.

#### 4.3.2 Dynamic development of the faculty

Developing multidisciplinary talent requires first breaking the path dependence of prioritizing theory over practice among university faculty, building a dual-qualified teaching team possessing both academic and practical capabilities. On one hand, “Scholar-in-Residence” positions should be established by inviting experts and scholars from health authorities, medical insurance bureaus, pharmaceutical enterprises, and high-level think tanks to regularly conduct teaching and research on campus, bringing the latest policy and industry developments into the classroom. On the other hand, formulate professional growth plans for faculty to ensure dynamic alignment between their professional development and the talent development framework. Firstly, strengthen interdisciplinary backgrounds of internal faculty by encouraging teachers from relevant departments, such as medical schools and business schools, to form interdisciplinary teaching teams. This fosters the collision and integration of ideas from educators in different disciplines. Secondly, support young faculty to enter international health governance organizations or prestigious overseas universities for training and exchange. This facilitates the acquisition of cutting-edge academic concepts and case resources, expanding global governance and innovation capabilities of the faculty (38). Thirdly, establish a multi-dimensional faculty evaluation mechanism centered on teaching competency, teaching outcomes, and project innovation (39). Provide special funding and talent incentives to top-rated teachers, motivating continuous innovation and high-quality teaching and research.

#### 4.3.3 Continuous professional development support mechanism for graduates

Facing the new situation of rapid iteration of medical technology, dynamic adjustment of health policies, and continuous upgrading of the health industry, graduates must possess the ability of lifelong learning. The talent development framework should build a future-oriented CPD support mechanism, extending school education across the full career trajectory (40). Universities should establish a “Health Economics Knowledge Update Cloud Platform” based on alumni networks. This platform could provide high-level online courses, electronic libraries, the latest academic trends, and case databases to graduates for life. Furthermore, it should focus on developing micro-credential courses covering emerging cross-disciplinary fields such as AI analysis for medical policies and digital health, meeting the updated requirements for graduates’ vocational skills and guaranteeing their competitiveness in the labor market. The CPD support mechanism encourages reciprocal feedback (41). Universities should regularly conduct surveys on alumni career trajectories and ability development. Authentic feedback from the frontline serves as a crucial basis for OBE, helping universities identify emerging shifts in industry talent demands. Simultaneously, inviting alumni, teachers, students, and industry experts on the same platform to form an interactive community. Encourage alumni to share frontier issues, typical cases, and policy challenges encountered in their work. This is a good way to enrich teaching case bases and drive the deep integration of talent development with industrial needs.

## 5 Discussion

This study analyzes the structural challenges of social security systems, treating the “human capital deficit” as a pivotal variable for the long-term governance efficiency of social security systems. Distinct from studies that focus on fiscal inputs or policy adjustments, we argue that the sustainability of social security depends on the competency of talent. By constructing a competency-based talent development framework in health economics, we provide an education reform pathway that combines theoretical depth with practical feasibility. The subsequent discussion elaborates on how this framework reconstructs the interdisciplinary education paradigm, extends its strategic implications to the sustainability of social security systems and health policies, and relies on a collaborative development mechanism for effective implementation.

### 5.1 Reconstructing the interdisciplinary education paradigm

Existing health economics education suffers from “disciplinary silos,” where universities fail to integrate economics, medicine, management, and law into a cohesive training scheme. This prevents students from forming a comprehensive and systemic vision to solve real-world health problems. To address this, we propose a “T-shaped” development model supported by a comprehensive curriculum paradigm. Through Outcome-Based Education, real-world simulation-based teaching, and progressive case study method, students can learn to view medical behaviors and economic cost not as isolated technical operations but as components of a complex socio-economic network. This cognitive reframing helps to rectify the Inverse Care Law, prompting future practitioners to prioritize the health rights of vulnerable populations and advocate for health equity throughout their careers.

### 5.2 Implications for social security systems and health policies

The proposed talent development framework extends beyond the scope of higher education and offers significant policy implications for the long-term sustainability of social security systems. First, it avoids the diminishing marginal returns of fiscal investment. International evidence suggests that in the absence of refined management, purely relying on fiscal expansion to increase medical insurance coverage or raise reimbursement ratios inevitably encounters the constraint of the law of diminishing marginal returns (42, 43). Without human capital intervention, new medical insurance funds are easily consumed by induced demand, over-medicalization, and inefficient allocation. Developing health economics talent will help capital investment translate into substantial health outcomes. Second, it improves resource allocation efficiency. Through systematic health economics education, future decision-makers can internalize the concepts of opportunity cost and evidence-based decision-making. Whether negotiating medical insurance directories in government medical insurance departments or conducting operational management in medical institutions, they possess the ability to apply HTA and cost-effectiveness analysis. This micro-level capability enhancement converges to drive a macro-level transformation of social security systems, thereby significantly improving public fund utility and resource allocation efficiency. Finally, it bolsters the resilience and long-term solvency of health policies. Facing payment pressures from population aging, cultivating professionals with a life-course management perspective is essential. This enables the formulation of forward-looking preventive policies, achieving intergenerational equity in resource distribution and guaranteeing the long-term viability of social security systems.

### 5.3 Collaborative development mechanism among the government, industry, universities, and research institutions

This study proposes a collaborative development mechanism, guided by the government, centered on universities, and supported by the active participation of the health industry and research institutions. In this mechanism, the policy requirements of social security departments, the critical challenges of the health industry, and the talent supply from universities achieve dynamic alignment. Through collaborative curriculum development, co-construction of practice bases, joint research projects, and the dual-mentor system, the time lag between talent development and social demand is significantly minimized. This ensures that human capital rapidly adapts to emerging trends, providing continuous intellectual support for the modernization of health governance.

## 6 Conclusion

Confronted with the structural challenges posed by an aging population, rising chronic disease burdens, and inequitable resource allocation, treatment-centric downstream interventions have become increasingly inadequate for contemporary health governance. Consequently, there is an urgent imperative to shift the strategic focus upstream to the supply of human capital. Human capital serves as a critical factor for maintaining the long-term solvency and service efficiency of social security systems. This study establishes a competency-based talent development framework and a “T-shaped” talent development model in health economics. Taking curriculum construction and pedagogical reform as the entry point, this study proposes a comprehensive curriculum paradigm that integrates knowledge, skills, and professional attributes, guided by the principles of demand orientation, competency drive, and systems thinking. It introduces innovative pedagogical methods, including Outcome-Based Education (OBE), real-world simulation-based teaching, and the progressive case study method. Furthermore, a collaborative development mechanism among the government, industry, universities, and research institutions is proposed to ensure the dynamic alignment of talent development with national strategies and industrial demands. Within this framework, graduates are equipped with interdisciplinary knowledge and practical competency to address complex challenges, including medical resource allocation and institutional innovation. This study provides an instructive and replicable educational paradigm for other interdisciplinary fields, offering a sustainable pathway to enhance the governance effectiveness and equity of social security systems.

## Data Availability

The original contributions presented in the study are included in the article/supplementary material, further inquiries can be directed to the corresponding author.

## Footnotes

1 United Nations, Department of Economic and Social Affairs, Population Division (2024). World Population Prospects: The 2024 Revision, custom data acquired via website. Available at: https://population.un.org/wpp/.

2 Global Burden of Disease Collaborative Network, Global Burden of Disease Study 2021 (GBD 2021) Results (2024, Institute for Health Metrics and Evaluation—IHME) https://www.who.int/news-room/fact-sheets/detail/noncommunicable-diseases.

3 Source of data: https://data.stats.gov.cn/easyquery.htm?cn=C01.

4 Source of data: https://data.who.int/countries/156.

## References

[1] Jaul E Barron J. Age-related diseases and clinical and public health implications for the 85 years old and over population. Front Public Health. (2017) 5. doi: 10.3389/fpubh.2017.00335, 29312916 PMC5732407

[2] Pan X-F Wang L Pan A. Epidemiology and determinants of obesity in China. Lancet Diabetes Endocrinol. (2021) 9:373–92. doi: 10.1016/S2213-8587(21)00045-0, 34022156

[3] Tudor Hart J. The inverse care law. Lancet. (1971) 297:405–12. 10.1016/s0140-6736(71)92410-x 4100731

[4] Cookson R Doran T Asaria M Gupta I Mujica FP. The inverse care law re-examined: a global perspective. Lancet. (2021) 397:828–38. doi: 10.1016/S0140-6736(21)00243-9, 33640069

[5] McMahon NE. Framing action to reduce health inequalities: what is argued for through use of the ‘upstream–downstream’ metaphor? J Public Health. (2022) 44:671–8. doi: 10.1093/pubmed/fdab157, 34056659 PMC9424054

[6] Lorenc T Petticrew M Welch V Tugwell P. What types of interventions generate inequalities? Evidence from systematic reviews. J Epidemiol Community Health. (2013) 67:190–3. doi: 10.1136/jech-2012-201257, 22875078

[7] Mills A. Leopard or chameleon? The changing character of international health economics. Trop Med Int Health. (1997) 2:963–77. 9357486 10.1046/j.1365-3156.1997.d01-159.x

[8] Barbu L. Global trends in the scientific research of the health economics: a bibliometric analysis from 1975 to 2022. Heal Econ Rev. (2023) 13:31. doi: 10.1186/s13561-023-00446-7, 37171506 PMC10176285

[9] Fuchs VR. The future of health economics. J Health Econ. (2000) 19:141–57. 10947574 10.1016/s0167-6296(99)00033-8

[10] Barrett JS. "Health economics and the healthcare industry" In: Fundamentals of drug development: Wiley (2022). 319–32. doi: 10.1002/9781119913276.ch17

[11] Hertling S Schöffski O Graul I Schleußner E. Digital health economics education: perspectives, potential and barriers at German medical universities. Front Med (Lausanne). (2025) 12, 1–2. doi: 10.3389/fmed.2025.1624347, 40823547 PMC12354404

[12] Folland S Goodman AC Stano M Danagoulian S. The economics of health and health care. New York: Routledge (2023).

[13] Pan A Liu L Wang C Guo H Hao X Wang Q . Association of public health interventions with the epidemiology of the COVID-19 outbreak in Wuhan, China. JAMA. (2020) 323:1915. doi: 10.1001/jama.2020.6130, 32275295 PMC7149375

[14] Jakovljevic M Chang H Pan J Guo C Hui J Hu H . Successes and challenges of China’s health care reform: a four-decade perspective spanning 1985—2023. Cost Eff Resour Alloc. (2023) 21:59. doi: 10.1186/s12962-023-00461-9, 37649062 PMC10469830

[15] Li L Fu H. China’s health care system reform: Progress and prospects. Int J Health Plann Manag. (2017) 32:240–53. doi: 10.1002/hpm.2424, 28612498

[16] Saxena S Amritesh Misra SC. Consumer value preferences in healthcare: insights for value-centred management. J Creating Value. (2021) 7:219–31. doi: 10.1177/23949643211042246

[17] Yang C-C. The integrated model of core competence and core capability. Total Qual Manag Bus Excell. (2015) 26:173–89. doi: 10.1080/14783363.2013.820024

[18] Pype P Mertens F Helewaut F Krystallidou D. Healthcare teams as complex adaptive systems: understanding team behaviour through team members’ perception of interpersonal interaction. BMC Health Serv Res. (2018) 18:570. doi: 10.1186/s12913-018-3392-3, 30029638 PMC6053823

[19] Adam T. Advancing the application of systems thinking in health. Health Res Policy Syst. (2014) 12:50. doi: 10.1186/1478-4505-12-50, 25160646 PMC4245197

[20] Trochim WM Cabrera DA Milstein B Gallagher RS Leischow SJ. Practical challenges of systems thinking and modeling in public health. Am J Public Health. (2006) 96:538–46. doi: 10.2105/AJPH.2005.066001, 16449581 PMC1470516

[21] Peters DH. The application of systems thinking in health: why use systems thinking? Health Res Policy Syst. (2014) 12:51. doi: 10.1186/1478-4505-12-51, 25160707 PMC4245196

[22] Rice T. The behavioral economics of health and health care. Annu Rev Public Health. (2013) 34:431–47. doi: 10.1146/annurev-publhealth-031912-114353, 23297657

[23] Kickbusch I Piselli D Agrawal A Balicer R Banner O Adelhardt M . The lancet and financial times commission on governing health futures 2030: growing up in a digital world. Lancet. (2021) 398:1727–76. doi: 10.1016/S0140-6736(21)01824-9, 34706260

[24] Dash S Shakyawar SK Sharma M Kaushik S. Big data in healthcare: management, analysis and future prospects. J Big Data (2019). 6:54. doi: 10.1186/s40537-019-0217-0

[25] Brown M Nic Giolla Mhichíl M Beirne E Mac Lochlainn C. The global micro-credential landscape: charting a new credential ecology for lifelong learning. J Learn Dev. (2021) 8:228–54. doi: 10.56059/jl4d.v8i2.525

[26] Pastorino R De Vito C Migliara G Glocker K Binenbaum I Ricciardi W . Benefits and challenges of big data in healthcare: an overview of the European initiatives. Eur J Pub Health. (2019) 29:23–7. doi: 10.1093/eurpub/ckz168, 31738444 PMC6859509

[27] Crown W Buyukkaramikli N Thokala P Morton A Sir MY Marshall DA . Constrained optimization methods in health services research—an introduction: report 1 of the ISPOR optimization methods emerging good practices task force. Value Health. (2017) 20:310–9. doi: 10.1016/j.jval.2017.01.013, 28292475

[28] Beauchamp TL. Methods and principles in biomedical ethics. J Med Ethics. (2003) 29:269–74. doi: 10.1136/jme.29.5.269, 14519835 PMC1733784

[29] Harden RM. Outcome-based education: the future is today. Med Teach. (2007) 29:625–9. doi: 10.1080/01421590701729930, 18236247

[30] Chen G Wang H Zhou L Yang J Xu L Liang Y. Development and applications of graduate outcome-based curriculum for basic medical education. Front Med (Lausanne). (2024) 11:2. doi: 10.3389/fmed.2024.1400811, 39219793 PMC11362034

[31] Pierson P. Increasing returns, path dependence, and the study of politics. Am Polit Sci Rev. (2000) 94:251–67. doi: 10.2307/2586011

[32] Savedoff WD de Ferranti D Smith AL Fan V. Political and economic aspects of the transition to universal health coverage. Lancet. (2012) 380:924–32. doi: 10.1016/S0140-6736(12)61083-6, 22959389

[33] Puri S. Teaching with the case method to build and teach management theory in offline and online courses. Innov Educ Teach Int. (2025):1–12. doi: 10.1080/14703297.2025.2482074

[34] Dollinger M Lodge J Coates H. Co-creation in higher education: towards a conceptual model. J Mark High Educ. (2018) 28:210–31. doi: 10.1080/08841241.2018.1466756

[35] Plewa C Galán-Muros V Davey T. Engaging business in curriculum design and delivery: a higher education institution perspective. High Educ. (2015) 70:35–53. doi: 10.1007/s10734-014-9822-1

[36] Helle L Tynjälä P Olkinuora E. Project-based learning in post-secondary education – theory, practice and rubber sling shots. High Educ. (2006) 51:287–314. doi: 10.1007/s10734-004-6386-5

[37] Galán-Muros V Plewa C. What drives and inhibits university-business cooperation in Europe? A comprehensive assessement. R D Manag. (2016) 46:369–82. doi: 10.1111/radm.12180

[38] Saroyan A Trigwell K. Higher education teachers’ professional learning: process and outcome. Stud Educ Eval. (2015) 46:92–101. doi: 10.1016/j.stueduc.2015.03.008

[39] Chalmers D. Progress and challenges to the recognition and reward of the scholarship of teaching in higher education. High Educ Res Dev. (2011) 30:25–38. doi: 10.1080/07294360.2011.536970

[40] Mandelid MB Dyngeland ES Teslo S Lerum Ø Tjomsland HE Jenssen ES . Workplace-based continuing professional development program for physically active learning: designing a framework and prospective directions. Front Educ. (2024) 9, 2–4. doi: 10.3389/feduc.2024.1407542

[41] Moore DE Green JS Gallis HA. Achieving desired results and improved outcomes: integrating planning and assessment throughout learning activities. J Contin Educ Heal Prof. (2009) 29:1–15. doi: 10.1002/chp.20001, 19288562

[42] Global spending on health 2020: Weathering the storm. Geneva: World Health Organization; (2020). 84 p.

[43] Yip W Fu H Chen AT Zhai T Jian W Xu R . 10 years of health-care reform in China: progress and gaps in universal health coverage. Lancet. (2019) 394:1192–204. doi: 10.1016/S0140-6736(19)32136-1, 31571602

